# “Spermidine restores dysregulated autophagy and polyamine synthesis in aged and osteoarthritic chondrocytes via EP300”

**DOI:** 10.1038/s12276-019-0224-4

**Published:** 2019-03-01

**Authors:** Rosa Maria Borzì, Silvia Cetrullo, Stefania D’Adamo, Manuela Minguzzi, Flavio Flamigni

**Affiliations:** 1 Laboratorio di Immunoreumatologia e Rigenerazione Tissutale, IRCCS Istituto Ortopedico Rizzoli, Bologna, Italy; 2 Dipartimento di Scienze Biomediche e Neuromotorie, Università di Bologna, Bologna, Italy; 3 Dipartimento di Scienze Mediche e Chirurgiche, Università di Bologna, Bologna, Italy

**Keywords:** Epigenetics, Experimental models of disease

In their recent article, Sacitharan et al.^1^ showed that spermidine restores dysregulated autophagy in chondrocytes and explored the underlying mechanisms, focusing on the role of EP300. We read the article with much interest since it matches our current research interests, in keeping with our multiple contributions at the International FEBS and OARSI Research Congresses since 2015.

However, we feel that the conclusions drawn by the authors do not properly take into account what has been published in the field. The authors correctly cited in the Introduction that “inhibition of EP300 by spermidine treatment may influence post-translational modification of essential autophagy-related protein complexes”, but later, in the Discussion, they state that “Spermidine has previously been shown to function (in part) via activation of the EP300 acetyltransferase”, referencing articles whose message is instead exactly the opposite, as also clearly evident in the title^2,3^.

EP300 is an acetyltransferase whose major substrates are H3, FOXO1, HDAC1, SIRT2, and ALX1^4^ and has a central role in many cell functions, acting as a transcriptional co-activator or a modulator of signaling pathways.

Eisenberg pointed at the hypoacetylation of histone H3 as a conserved mechanism whereby spermidine promotes longevity and concluded that “The altered acetylation status of the chromatin led to significant upregulation of various autophagy-related transcripts, triggering autophagy in yeast, flies, worms and human cells”^2^. Pietrocola elegantly showed that spermidine inhibits EP300 activity in cell free systems, and that specific knockdown of EP300 leads to increased LC3 and decreased p62 signal and therefore increased autophagy^3^, and concluded that “EP300 acts as an endogenous repressor of autophagy and that potent autophagy inducers including spermidine de facto act as EP300 inhibitors”.

Given that spermidine acts as a competitive inhibitor of EP300 activity^3^, it is conceivable that it also leads to increased EP300 gene and protein expression. However, the available literature contradicts the hypothesis of the authors: “inhibition” and not “activation” of EP300 and its acetylating activity on critical autophagy proteins has been connected with the rescue of autophagy since 2009^5^ and further reviewed in ref. ^6^. Several papers have been published in the field that almost invariably state that acetylation of autophagy-related proteins depresses autophagy^7^. A more recent paper specifically shows that beclin-1 acetylation reduces autophagosome formation^8^. In the figure titled “Spermidine activates chondrocyte autophagy via EP300”, panels E and G would indicate that EP300 siRNA depresses autophagy, in keeping with the reduced expression of Beclin1 and LC3 II:I. However, the correct interpretation of this information would require the use of lysosome inhibitors, because an enhancement of the autophagic flux leads to the reduced expression of proteins that are disposed of in autophagolysosomes^9^.
